# Supplementary material for: A single 2′-O-methylation of ribosomal RNA gates assembly of a functional ribosome
Source: Nat Struct Mol Biol. 2022 Dec 19;30(1):91–8. doi: 10.1038/s41594-022-00891-8 (PMC9851907; doi:10.1038/s41594-022-00891-8)
Supplement: Supplementary file 1 — Reporting Summary [file 41594_2022_891_MOESM1_ESM.pdf]

nature portfolio

Corresponding author(s): Arlen Johnson and David Taylor

Last updated by author(s): Oct 27, 2022

Reporting Summary

Nature Portfolio wishes to improve the reproducibility of the work that we publish. This form provides structure for consistency and transparency in reporting. For further information on Nature Portfolio policies, see our Editorial Policies and the Editorial Policy Checklist.

Statistics

For all statistical analyses, confirm that the following items are present in the figure legend, table legend, main text, or Methods section.

n/a	Confirmed	
☐	☒	The exact sample size (*n*) for each experimental group/condition, given as a discrete number and unit of measurement
☐	☒	A statement on whether measurements were taken from distinct samples or whether the same sample was measured repeatedly
☐	☒	The statistical test(s) used AND whether they are one- or two-sided<br>*Only common tests should be described solely by name; describe more complex techniques in the Methods section.*
☒	☐	A description of all covariates tested
☒	☐	A description of any assumptions or corrections, such as tests of normality and adjustment for multiple comparisons
☒	☐	A full description of the statistical parameters including central tendency (e.g. means) or other basic estimates (e.g. regression coefficient) AND variation (e.g. standard deviation) or associated estimates of uncertainty (e.g. confidence intervals)
☐	☒	For null hypothesis testing, the test statistic (e.g. F, t, r) with confidence intervals, effect sizes, degrees of freedom and P value noted<br>*Give P values as exact values whenever suitable.*
☒	☐	For Bayesian analysis, information on the choice of priors and Markov chain Monte Carlo settings
☒	☐	For hierarchical and complex designs, identification of the appropriate level for tests and full reporting of outcomes
☒	☐	Estimates of effect sizes (e.g. Cohen's *d*, Pearson's *r*), indicating how they were calculated

Our web collection on statistics for biologists contains articles on many of the points above.

Software and code

Policy information about availability of computer code

Data collection	Data were collected on an FEI Titan Krios microscope with a K3 direct electron detector using Serial EM v3.8.
Data analysis	Cyber T webserver was used to analyze mass spectrometry data. Cryo-EM data was analyzed using cryoSPARC (Live and v3.2) and RELION (v3.1.3). The model was built using COOT v1.0 and relaxed using flexible molecular dynamics fitting with ISOLDE v1.4, then finally subjected to real-space refinement as implemented in PHENIX v1.19.2. All molecular graphics were prepared with UCSF ChimeraX v1.0.

For manuscripts utilizing custom algorithms or software that are central to the research but not yet described in published literature, software must be made available to editors and reviewers. We strongly encourage code deposition in a community repository (e.g. GitHub). See the Nature Portfolio guidelines for submitting code & software for further information.

Data

Policy information about availability of data

All manuscripts must include a data availability statement. This statement should provide the following information, where applicable:

- Accession codes, unique identifiers, or web links for publicly available datasets
- A description of any restrictions on data availability
- For clinical datasets or third party data, please ensure that the statement adheres to our policy

The raw sequencing data are deposited in the Sequencing Read Archive (SRA) with accession number PRJNA892951. The structure of the Nog2-3xFLAG unmodified pre-60S subunit and its associated atomic coordinates have been deposited into the Electron Microscopy Data Bank (EMDB) and the Protein Data Bank (PDB) as EMDB-26485, and PDB 7UG6, respectively. All other data are available in the manuscript or supplementary materials. Requests for strains or plasmids will be fulfilled by the lead contact author, AWJ, upon request.

Field-specific reporting

Please select the one below that is the best fit for your research. If you are not sure, read the appropriate sections before making your selection.

☒ Life sciences ☐ Behavioural & social sciences ☐ Ecological, evolutionary & environmental sciences

For a reference copy of the document with all sections, see nature.com/documents/nr-reporting-summary-flat.pdf

Life sciences study design

All studies must disclose on these points even when the disclosure is negative.

Sample size	The number of particles used were limited by the amount of time that data could be collected.
Data exclusions	No data was excluded.
Replication	Genetic screens, biochemical assays, and cell imaging are representative of two independent experiments. Multiple structures were obtained at various time points that resembled one another.
Randomization	No randomization was performed. Randomization was not relevant to this study because it didnot involve human subjects or live animals where bias is possible.
Blinding	No blinding was performed. Blinding is not relevant to this study because it did not involve human subjects or liver animals where bias is possible.

Reporting for specific materials, systems and methods

We require information from authors about some types of materials, experimental systems and methods used in many studies. Here, indicate whether each material, system or method listed is relevant to your study. If you are not sure if a list item applies to your research, read the appropriate section before selecting a response.

Materials & experimental systems

n/a	Involved in the study	
☐	☒	Antibodies
☒	☐	Eukaryotic cell lines
☒	☐	Palaeontology and archaeology
☒	☐	Animals and other organisms
☒	☐	Human research participants
☒	☐	Clinical data
☒	☐	Dual use research of concern

Methods

n/a	Involved in the study	
☒	☐	ChIP-seq
☒	☐	Flow cytometry
☒	☐	MRI-based neuroimaging

Antibodies

Antibodies used	Primary antibodies used included rabbit anti-G6PD and rat anti-FLAG (Agilent). Secondary antibodies included goat anti-rabbit 680RD and goat anti-rat 800CW.
Validation	Primary antibodies have been verified by relative expression and knockdown to confirm specificity.
