# Supplementary figures and images for: A single 2′-O-methylation of ribosomal RNA gates assembly of a functional ribosome
Source: Nat Struct Mol Biol. 2022 Dec 19;30(1):91–8. doi: 10.1038/s41594-022-00891-8 (PMC9851907; doi:10.1038/s41594-022-00891-8)

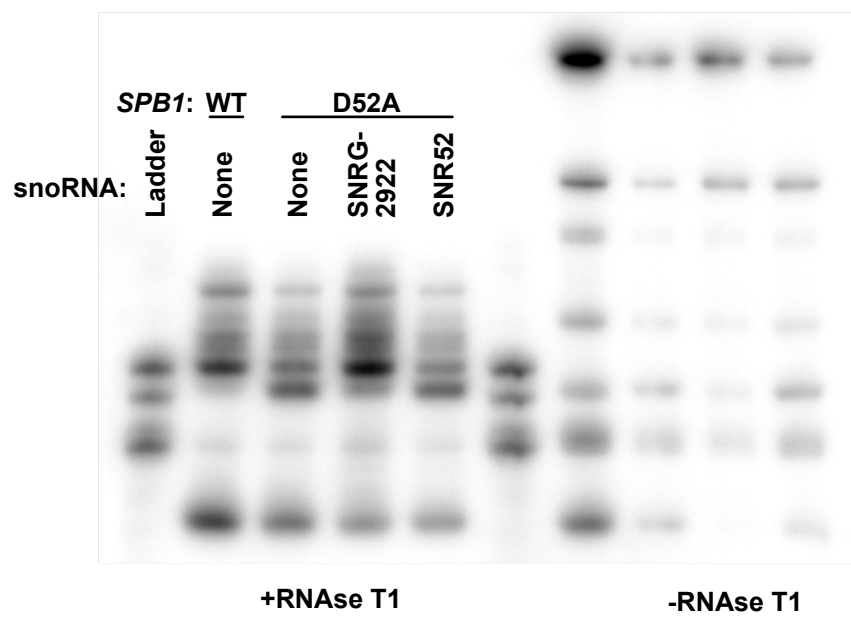

Supplement: Source Data Fig. 2 — Unprocessed gel. [file 41594_2022_891_MOESM5_ESM.pdf]

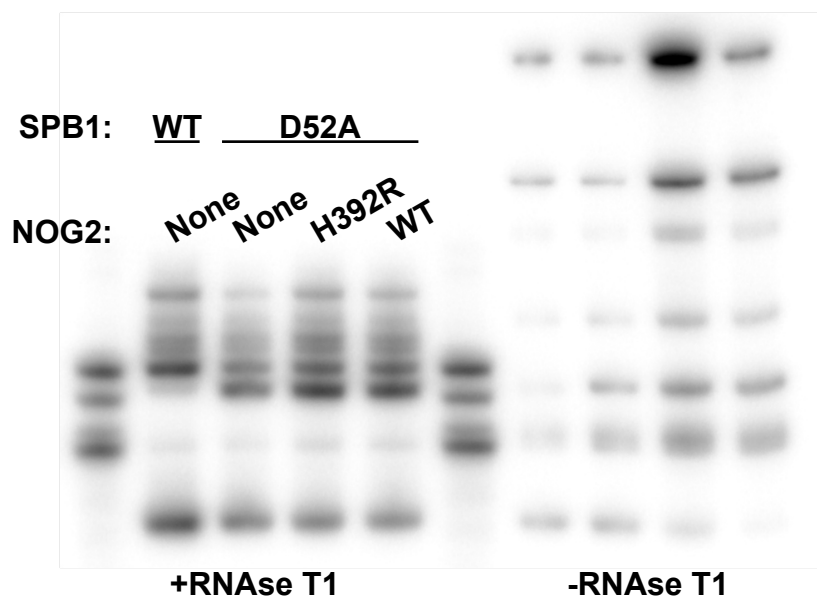

Supplement: Source Data Fig. 3 — Unprocessed gel. [file 41594_2022_891_MOESM6_ESM.pdf]

NOG2-3xFLAG

Vector WT S208A R389S S/R double

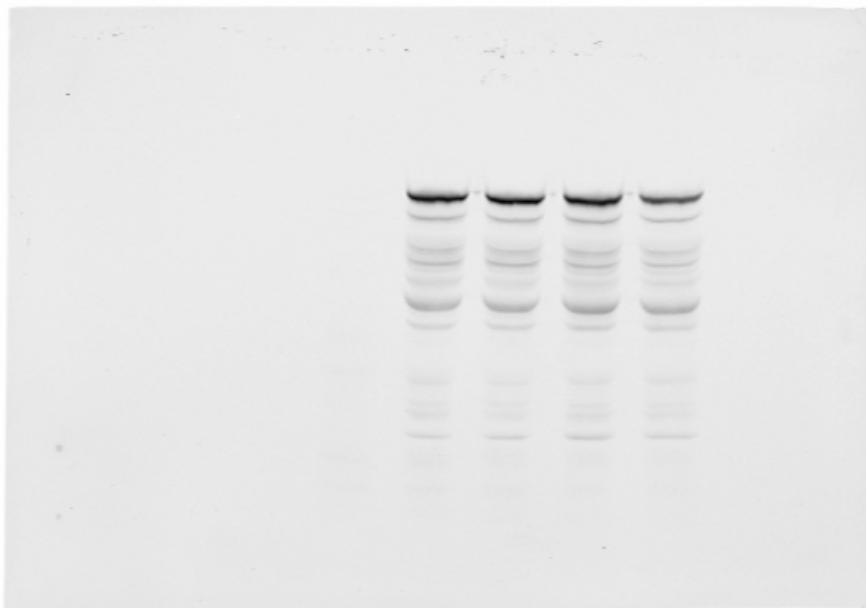

$\alpha$ FLAG

NOG2-3xFLAG

Vector WT S208A R389S S/R double

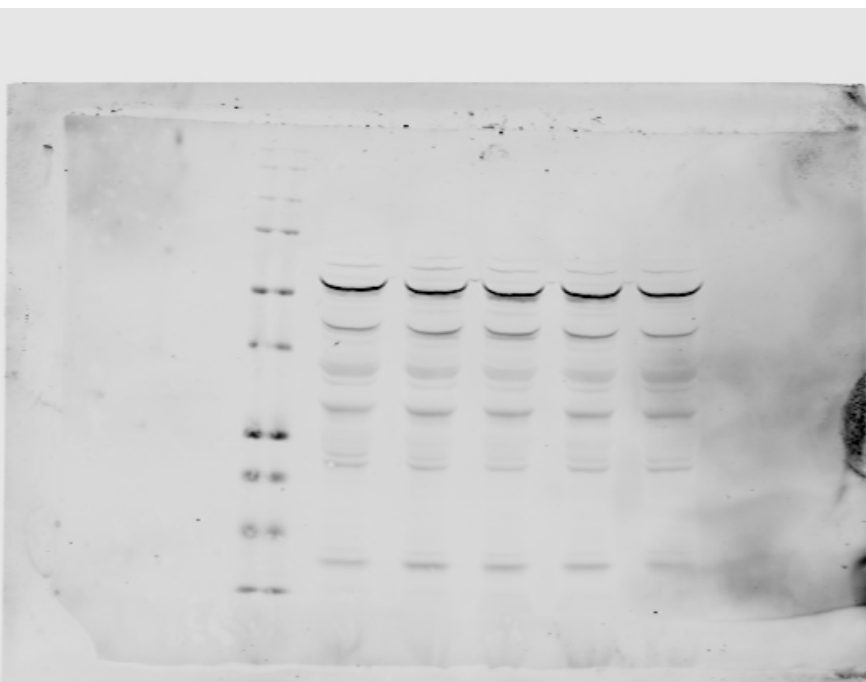

$\alpha$ G6PD

Supplement: Source Data Extended Data Fig. 2 — Unprocessed western blot scans. [file 41594_2022_891_MOESM8_ESM.pdf]

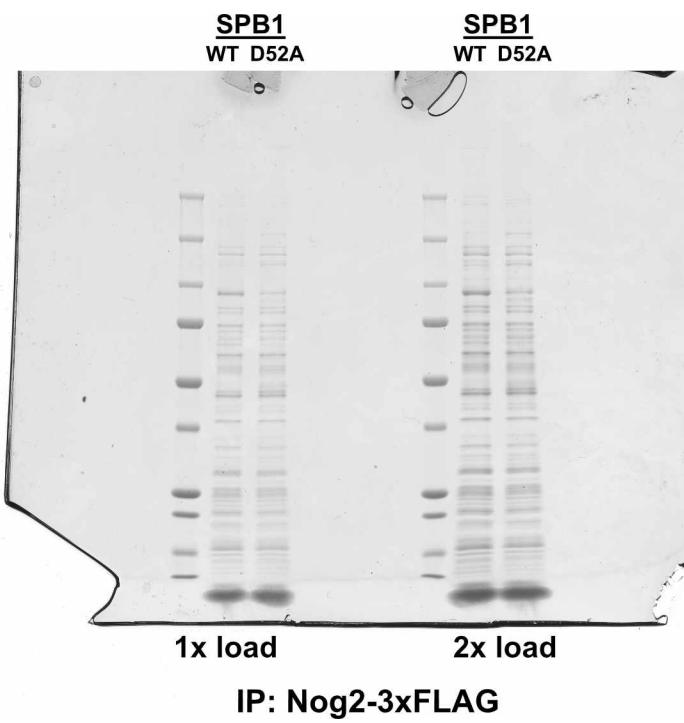

Supplement: Source Data Extended Data Fig. 4 — Unprocessed gel. [file 41594_2022_891_MOESM10_ESM.pdf]
